# Fasciotens^®^ for abdominal wall closure: current evidence and clinical perspectives

**DOI:** 10.1007/s10029-026-03793-6

**Published:** 2026-07-14

**Authors:** Melania Rivano, Noemi Tatti, Francesco Filidoro, Arianna Cadeddu, Adolfo Pisanu, Mauro Podda

**Affiliations:** 1 Hospital Pharmacy Unit, Cagliari University Hospital, Cagliari, Italy; 2 Department of Biomedical Sciences, School of Specialization in Clinical Pharmacology and Toxicology, University of Cagliari, Cagliari, Italy; 3 Hospital Pharmacy Unit, Mater Olbia Hospital, Olbia, Italy; 4 Department of General and Emergency Surgery, Cagliari University Hospital, Cagliari, Italy

**Keywords:** Narrative review, Open abdomen, Abdominal wall closure, Fascial traction, Ventral hernia, Fasciotens^®^

## Abstract

**Purpose:**

Management of open abdomen (OA) and complex ventral hernias remains associated with high morbidity, prolonged hospitalization, and substantial healthcare costs. Vertical fascial traction has emerged as a novel approach aimed at facilitating early primary fascial closure. This study provides a narrative review and multidimensional overview of the available evidence regarding the Fasciotens^®^ device, focusing on clinical outcomes, safety, economic considerations, and organizational aspects.

**Methods:**

A narrative systematic literature review was performed to identify studies evaluating the use of Fasciotens^®^ in adult patients with open abdomen or complex ventral hernias. Clinical, economic, organizational, and ethical domains were assessed.

**Results:**

Available evidence, primarily derived from observational studies and case series, suggests that the use of Fasciotens^®^ is associated with high rates of primary fascial closure, shorter time to closure, and reductions in intra‑abdominal pressure. Device‑related adverse events appear to be infrequent and mainly limited to minor skin‑related complications. Preliminary economic considerations indicate that earlier closure and avoidance of complex reconstructive procedures may reduce operative time, intensive care unit stay, and overall hospital resource utilization.

**Conclusion:**

Current evidence supports the clinical feasibility and potential value of Fasciotens^®^ as an adjunctive technology for the management of open abdomen and complex ventral hernias. However, the available data are limited by small sample sizes and non‑comparative study designs. Further prospective and comparative studies are needed to better define its role within standardized treatment pathways.

## Introduction

Management of abdominal wall defects, particularly in the context of open abdomen (OA) and complex ventral hernias, represents a major clinical and organizational challenge in modern surgery [1]. These conditions are frequently associated with severe underlying pathologies such as abdominal sepsis, trauma, ischemia, or previous multiple surgical interventions, and are characterized by high morbidity, prolonged intensive care unit (ICU) stay, increased risk of complications, and substantial healthcare costs [2].

The OA strategy is widely adopted in critically ill patients when primary fascial closure is contraindicated due to physiological instability, uncontrolled intra-abdominal sepsis, or the presence of abdominal compartment syndrome. While OA can be life-saving, prolonged exposure of the abdominal contents inevitably leads to progressive fascial retraction, loss of abdominal domain, and a marked reduction in the likelihood of achieving delayed primary fascial closure. Failure to restore fascial continuity is associated with the development of complex incisional hernias, entero-atmospheric fistulas, repeated surgical procedures, and long-term impairment in quality of life [3–5]. Similarly, complex ventral hernias, often defined by large defect size, loss of domain, previous failed repairs, and significant patient comorbidities, require advanced reconstructive techniques that are expensive and burdened by relevant complication rates [6]. Procedures such as component separation techniques or transversus abdominis release (TAR), although effective, are associated with prolonged operative time, increased surgical site occurrences, and higher overall costs, particularly when biological meshes are required [7].

Over the past decade, several strategies have been proposed to prevent fascial retraction and facilitate delayed primary closure in OA, including negative pressure wound therapy, mesh-mediated fascial traction, temporary abdominal closure systems, and pharmacological muscle relaxation using botulinum toxin type A [8–11]. Despite these advances, achieving early and tension-free fascial closure remains challenging, and no single approach has emerged as a universally accepted standard of care.

**Fig. 1:**
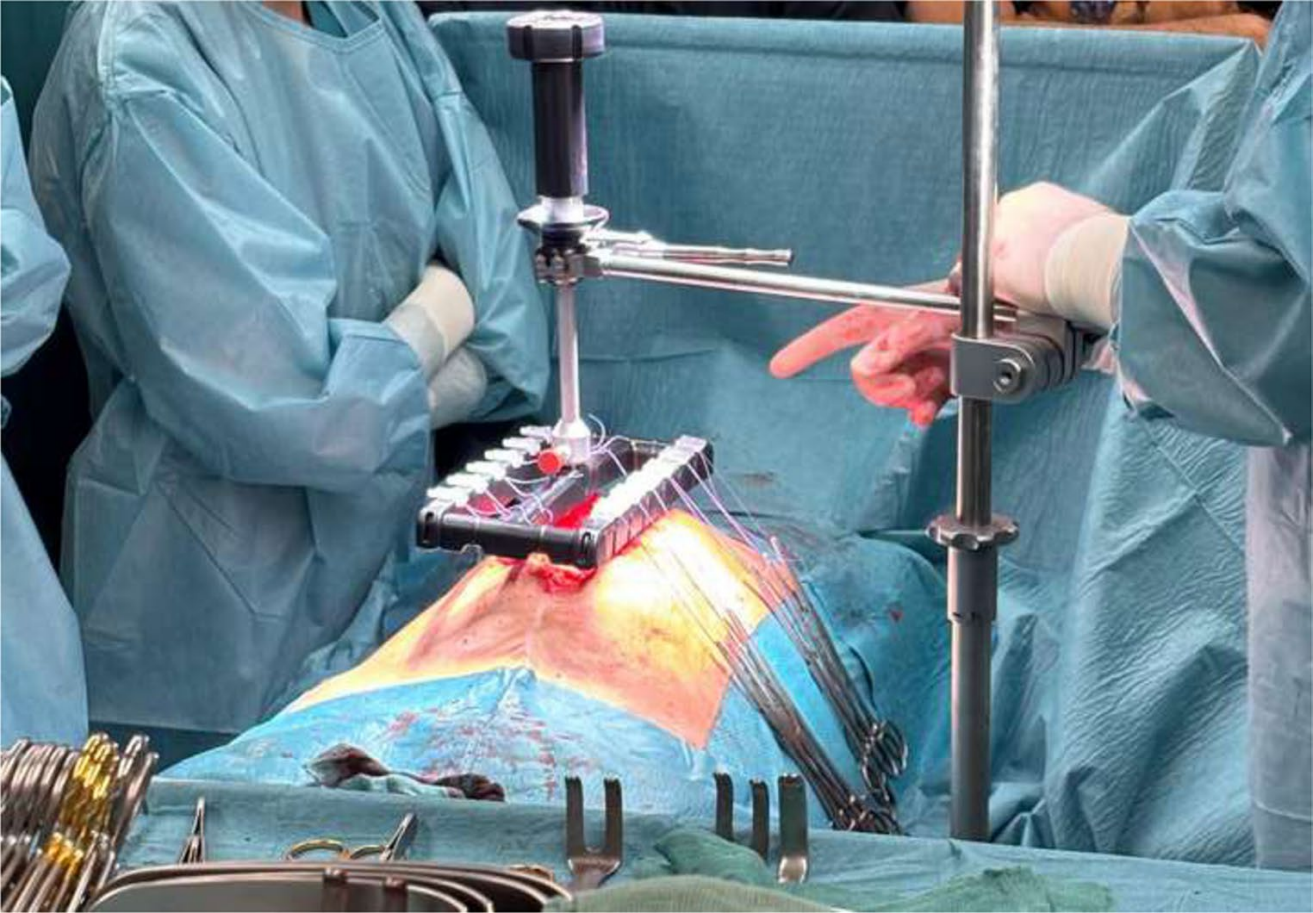
Intraoperative application of the Fasciotens^®^ vertical fascial traction system. The device applies controlled cranio-caudal traction through multiple fixation points, allowing progressive medialization and approximation of the fascial edges during abdominal wall closure

Vertical fascial traction represents a novel biomechanical method aimed at counteracting lateral fascial retraction by applying controlled, quantifiable forces along the cranio-caudal axis of the abdominal wall. The intraoperative application of the device is illustrated in Fig. 1. Fasciotens^®^ is currently the only commercially available device specifically designed to deliver continuous or intraoperative vertical fascial traction in patients with OA or complex ventral hernias. Preliminary clinical experience suggests that this approach may increase rates of primary fascial closure, reduce intra-abdominal pressure, and limit the need for extensive reconstructive procedures [12–13].

Given the emerging nature of the technology and the limited availability of comparative studies, this work aims to provide a narrative synthesis of the current evidence. This study provides a narrative review and multidimensional overview of the Fasciotens^®^ vertical fascial traction system for the management of open abdomen and complex ventral hernias. This assessment focuses on clinical, economic, organizational, and ethical aspects relevant to surgical practice.

## Methods

A structured literature review was conducted to provide a narrative synthesis of the available evidence. Although domains commonly included in Health Technology Assessment (HTA) frameworks (clinical, economic, organizational, and ethical aspects) were considered, the analysis was not designed as a formal HTA due to the lack of comparative and high-quality evidence. The assessment was designed to support evidence-informed decision making regarding the adoption of the Fasciotens^®^ vertical fascial traction system in the management of open abdomen and complex ventral hernias at the Emergency Surgery Department of Cagliari University Hospital (Cagliari Italy).

### Literature search strategy

A structured narrative literature review was performed to identify available evidence on the use of Fasciotens^®^ in patients with open abdomen or complex ventral hernias. Electronic searches were conducted on 15 January 2026 in PubMed including MEDLINE e PubMed Central (PMC). The search strategy employed the string (“Open abdomen” OR “Hernia”) AND (“Fasciotens^®^”). Additional studies were identified through manual screening of reference lists from relevant publications. Both indexed and non-indexed peer-reviewed articles were considered eligible, given the limited availability of high-level evidence for this emerging technology. Two independent reviewers screened titles and abstracts for eligibility.

### Eligibility criteria

Studies were included if they reported clinical outcomes related to the use of Fasciotens^®^ in adult patients with OA or complex ventral hernias. Eligible study designs included prospective and retrospective observational studies, case series, and case reports. Preclinical studies, pediatric applications, technical descriptions without clinical outcomes, and studies evaluating alternative traction systems were excluded. The review was conducted according to the PRISMA 2020 reporting guidelines [14].

### Data extraction and synthesis

Data were extracted on study design, patient population, clinical setting, duration of traction, rates of primary fascial closure, time to closure, complications, and follow-up outcomes. Given the heterogeneity of study designs and outcome measures, a quantitative meta-analysis was not feasible. Results were therefore synthesized descriptively and stratified according to clinical indication (open abdomen versus complex ventral hernia repair).

### Assessment domains

Clinical effectiveness was evaluated based on rates of primary fascial closure, reduction in fascial gap, time to definitive closure, and need for additional reconstructive techniques. Safety assessment focused on device-related adverse events, surgical site occurrences, and systemic complications. Economic evaluation considered direct device costs, operative time, length of ICU and hospital stay, and potential cost offsets associated with reduced complications or avoidance of complex procedures. Organizational impact was assessed in terms of workflow integration, training requirements, and resource utilization. Ethical and social aspects included considerations of equity of access, proportionality of benefit, and patient-centered outcomes.

## Results

**Fig. 2:**
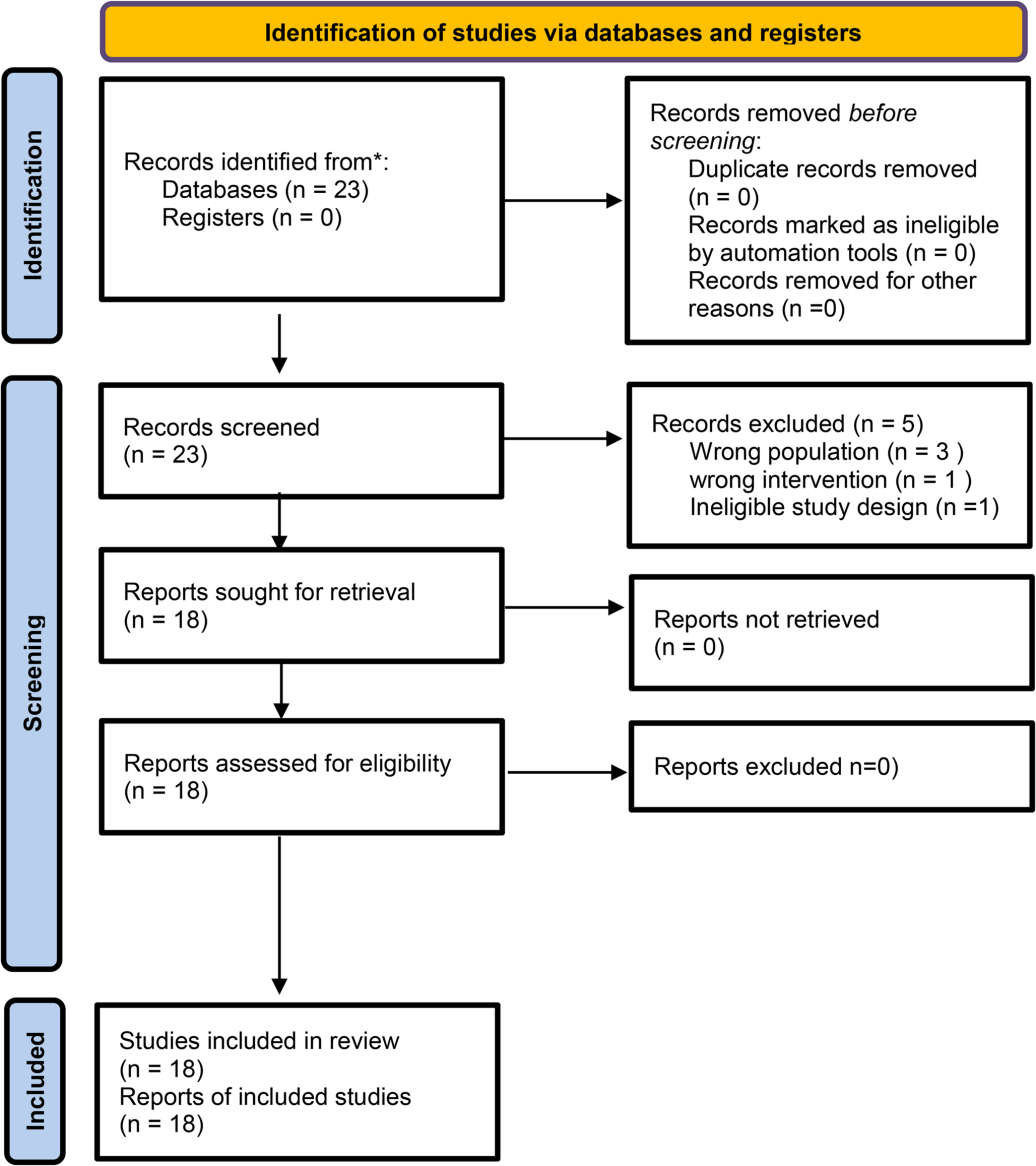
PRISMA flow diagram of the study selection process

Figure 2 summarizes the literature selection process according to the PRISMA flow diagram. The database search yielded 23 records. Following title and abstract screening, 18 articles underwent full-text evaluation and were subsequently included in the qualitative synthesis. Of these, 9 studies focused on Fasciotens Abdomen^®^ [12, 15–22], and 9 on Fasciotens Hernia^®^ [13, 23–30].

Eucker D et al. was excluded because Fasciotens^®^ was not used (wrong intervention) [31]. The study by Ziegler et al. was excluded because it concerns the application of Fasciotens^®^ Pediatric for newborns and infants diagnosed with a congenital or acquired abdominal wall defect, which is not the subject of the analysis [32]. Finally, the preclinical study [33] and the study by Niebuhr were excluded, as it was demonstrated that intraoperative fascial traction (IFT) causes significant medialisation of the anterior sheath of the rectus muscle in a cadaveric model [34]. The study by Capoccia Giovannini et al. [35] was excluded as it is a methodological protocol for the future European Hernia Society (EHS) guideline on ventral/incisional hernias in emergency situations. Although it mentions IFT and Fasciotens techniques as emerging and promising approaches, it does not present original interventional data.

Tables 1 and 2 summarise the characteristics of the included studies, with Table 1 focusing on Fasciotens^®^ Abdomen and Table 2 on Fasciotens^®^ Hernia.

**Table 1:** Characteristics of studies evaluating Fasciotens^®^ abdomen

Author (year)	Design	*N*	Follow-up / time to closure	Intervention	Key outcomes
Fung 2022 [12]	Multicenter retrospective	20	~ 7 d	Fasciotens^®^	PFC 100%; gap ~ 15→10 cm at 48 h
Nguyen 2024 [15]	Case report	1	14 d	AbThera + BTX-A + Fasciotens^®^	PFC; gap 18→5 cm; no complications
Hees 2020 [16]	Case report	1	6 d	Fasciotens^®^	PFC 12→4 with mesh; rapid closure
Miranda 2023 [17]	Case report	1	10 d	Vertical traction + BTX-A + NPWT	FTF 16→8 cm at 48 h; DFC 90.7%
Dohmen 2024 [18]	Consecutive case series	9	9 ± 3 d	Fasciotens^®^	PFC 6/9; IAP ↓; minor skin events
Mones 2024 [19]	Prospective series	9	~ 6 d	IFT + NPWT ± mesh	DFC 78%; no SSI/dehiscence
Mavc 2023 [20]	Case report	1	6 d	Fasciotens^®^ + AbThera^®^	Gap 15→0–1 cm; no device-related events
Fung 2019 [21]	Case report	1	14 d	Fasciotens^®^	Gap 15→10 cm; early closure; no major complications
Ioannidis 2019 [22]	Prospective observational study	13	9 d	Fasciotens + NPWT	DFC 84,62%

Abbreviations: *PFC* primary fascial closure, *DFC* definitive fascial closure, *NPWT* negative pressure wound therapy, *IFT* intraoperative fascial traction, *BTX-A* botulinum toxin A, *IAP* intra-abdominal pressure, *SSI* surgical site infection, *FU* follow-up, *NA* not available

**Table 2:** Characteristics of studies evaluating Fasciotens^®^ hernia

Author (year)	Design	*N*	Follow-up / time to closure	Intervention	Key outcomes
Niebuhr 2021 [13]	Prospective observational study	21	6–12 mo	IFT + BTX-A	PFC 95%; recurrence < 5%
De Matteis 2023 [23]	Case report	1	–	IFT + biological mesh	Improved primary closure; tension reduction
Quiroga-Centeno 2024 [24]	Scoping review	–	–	–	IFT/Fasciotens cited; limited high-level evidence
Niebuhr 2022 [25]	Multicenter prospective	50	6–12 mo	IFT	PFC > 90%; low SSI; no ACS
Niebuhr 2024 [26]	Retrospective	143	6 mo	IFT + BTX-A	Mean gap reduction 9.8 cm;
Woeste 2025 [27]	Prospective follow-up	100	12–24 mo	IFT (87% BTX-A)	Recurrence < 5%; SSO 33%
Bloemendaal 2024 [28]	Case report	3	Short-term	combination of RAWS and IFT	Feasible and safe
Balachandran 2025 [29]	Case report	1		IFT + BTx-A	Total gain in length 11.31 cm
Atici 2026 [30]	Video vignette				

Abbreviations: *ACS* Abdominal Compartment Syndrome, *PFC* primary fascial closure, *IFT* intraoperative fascial traction, *BTX-A* botulinum toxin A, *SSO* surgical site occurrence, *RAWS* robot-assisted abdominal wall surgery

### Clinical effectiveness in open abdomen

The available clinical evidence on the use of Fasciotens^®^ in the management of open abdomen is limited but consistent across published observational studies and case series. Fasciotens^®^ has been primarily applied in critically ill patients with severe abdominal sepsis, trauma, ischemia, or abdominal compartment syndrome, in whom early primary fascial closure was not feasible at the index operation. Across the identified studies, the application of vertical fascial traction was associated with high rates of delayed primary fascial closure, frequently exceeding 85–90% in selected patient cohorts [12, 18]. Fasciotens^®^ facilitated closure within relatively short timeframes reported in the available literature. Reported time to definitive closure ranged from intraoperative application lasting a few hours to continuous traction protocols maintained for several days in the ICU. In addition to improved closure rates, several studies reported a reduction in intra-abdominal pressure during traction, potentially reducing the risk of recurrent abdominal compartment syndrome. Fasciotens^®^ was often used in combination with negative pressure wound therapy, suggesting good compatibility with established open abdomen management strategies

### Clinical effectiveness in complex ventral hernia repair

Evidence regarding the use of Fasciotens^®^ in complex ventral hernia repair mainly derives from elective surgical settings involving patients with large abdominal wall defects, loss of domain, multiple previous failed repairs, and significant comorbidities. In this context, vertical fascial traction has been applied intraoperatively to achieve medialization of the rectus muscles and to reduce fascial tension prior to definitive closure [26] Reported outcomes suggest that the use of Fasciotens^®^ may reduce the need for advanced component separation techniques, such as TAR, in selected patients. Surgeons consistently reported improved fascial compliance and enhanced ability to achieve midline closure without excessive tension. Although long-term recurrence data remain limited, early postoperative outcomes indicate acceptable durability of repair when the device is integrated into standardized reconstructive pathways [27].

### Safety

Overall, Fasciotens^®^ demonstrated a favourable safety profile across both emergency and elective applications. Reported adverse events were predominantly minor and mainly related to skin pressure at device contact points, including transient erythema, superficial skin lesions, or localized discomfort [18, 26]. These events were generally self-limiting and resolved without long-term consequences. No severe device-related complications, such as fascial tearing, neurovascular injury, or device failure, were consistently reported. Importantly, the application of controlled vertical traction did not appear to increase the incidence of entero-atmospheric fistulas or surgical site infections when compared with conventional management strategies. Nevertheless, interpretation of safety outcomes is limited by the observational nature and small sample sizes of the available studies.

### Economic impact

Formal cost-effectiveness analyses of Fasciotens^®^ are currently lacking. However, publicly available data provide an estimate of the acquisition costs. According to the National Institute for Health and Care Excellence, the estimated acquisition cost of Fasciotens^®^ Abdomen is approximately £3,995 per single-use device, while Fasciotens^®^ Hernia costs around £1,760, with an additional reusable carrier priced at £3,650 [36]. These figures provide a reference for technology costs; however, actual prices may vary across healthcare systems and procurement agreements. Importantly, these estimates do not account for potential cost offsets associated with earlier fascial closure, reduced operative time, shorter intensive care unit stay, and avoidance of complex reconstructive procedures. Indirect economic considerations based on reported clinical outcomes and resource utilization suggest that the technology may offer potential cost savings. These include reductions in operative time, shorter ICU and overall hospital length of stay, and decreased need for complex reconstructive procedures or expensive biological meshes [13, 16].

In the context of OA management, earlier achievement of fascial closure may reduce the number of reoperations and long-term costs associated with the development of large incisional hernias. Fasciotens Abdomen^®^ is proposed as a technology that could reduce complications in certain indications; however, direct economic evidence remains limited, and NICE recommends larger comparative studies [36]. In elective ventral hernia repair, simplification or avoidance of component separation techniques may further contribute to reduced perioperative morbidity and associated healthcare expenditures.

### Organizational, ethical, and social aspects

From an organizational perspective, Fasciotens^®^ can be integrated into existing surgical workflows with relatively limited additional training requirements [27]. The possibility of both intraoperative and ICU application provides flexibility in patient management and may contribute to more efficient use of operating room and critical care resources. Ethical and social considerations include equitable access to innovative surgical technologies and appropriate allocation of resources in high-cost clinical scenarios. By potentially reducing morbidity, improving functional recovery, and limiting long-term complications, Fasciotens^®^ may contribute to improved quality of life and align with principles of patient-centered and value-based care.

## Discussion

This structured narrative review valuated the clinical, economic, organizational, and ethical implications of the Fasciotens^®^ vertical fascial traction system in the management of OA and complex ventral hernias. Overall, the findings suggest that Fasciotens^®^ represents a promising adjunctive technology capable of addressing some of the most critical challenges associated with abdominal wall closure in high-risk surgical settings. From a clinical perspective, the available evidence indicates that vertical fascial traction may significantly improve rates of delayed primary fascial closure in OA patients. By counteracting progressive lateral fascial retraction, Fasciotens^®^ appears to preserve abdominal wall compliance and facilitate earlier restoration of fascial continuity. This is a clinically relevant outcome, as failure to achieve primary closure is strongly associated with increased morbidity, prolonged hospitalization, development of complex incisional hernias, and long-term impairment in quality of life.

In elective complex ventral hernia repair, the intraoperative application of vertical traction may reduce the need for extensive component separation techniques, such as TAR. While these techniques remain effective, they are associated with increased operative complexity, higher rates of surgical site occurrences, and substantial resource utilization. The potential of Fasciotens^®^ to simplify reconstructive strategies in selected patients aligns with current efforts to reduce surgical invasiveness while maintaining durable outcomes. When compared with existing strategies for OA management, including negative pressure wound therapy alone or mesh-mediated fascial traction, Fasciotens^®^ introduces a distinct biomechanical approach based on controlled cranio-caudal force application. This mechanism allows for quantifiable and adjustable traction, which may enhance safety and reproducibility. Importantly, available evidence suggests that the device can be safely combined with established techniques, supporting its role as a complementary rather than substitutive technology.

Safety outcomes reported in the literature are encouraging, with predominantly minor and reversible adverse events. The absence of consistently reported severe device-related complications supports the feasibility of vertical fascial traction in both emergency and elective contexts. However, the limited number of patients and the observational nature of the evidence necessitate cautious interpretation, particularly regarding rare or long-term complications.

From an economic standpoint, although formal cost-effectiveness analyses are currently unavailable, indirect evidence suggests that Fasciotens^®^ may contribute to more efficient resource utilization. Earlier fascial closure, reduced need for complex reconstructions, and shorter intensive care and hospital stays are all factors that may translate into meaningful cost savings at the institutional level. These considerations are particularly relevant in healthcare systems facing increasing financial constraints and growing demand for high-cost surgical care.

Organizationally, the integration of Fasciotens^®^ into hospital workflows appears feasible, with limited training requirements and flexibility of use across different clinical settings. The potential reduction in length of stay and critical care utilization may further support hospital capacity management and improve patient flow.

This review has several important limitations. First, the available evidence is predominantly derived from observational studies, case series, and case reports, with a notable lack of randomized or comparative studies. In addition, most studies included small sample sizes and non-comparative designs, limiting the strength of the conclusions. Second, the literature search was restricted to a single database, and no formal quality assessment of the included studies was performed. Third, substantial heterogeneity across patient populations, clinical indications, treatment protocols, and outcome measures reduces the comparability of reported results and limits the ability to draw definitive conclusions regarding comparative effectiveness. Finally, the inclusion of both open abdomen and elective ventral hernia settings may further limit the generalizability of the findings. These limitations are also reflected in the currently available evidence regarding patient-reported outcomes and quality of life (QoL). The study conducted by Woeste et al. was the first to investigate quality of life (QoL) in ventral hernia repair using the IFT, in which the HerQles questionnaire was employed [27]. In the study, all patients completed the HerQles questionnaire during the follow-up visit. In the analyzed cohort, the mean HerQles Summary Score was 68.47 ± 16.3 (mean ± SEM). A limitation of the study by Woeste et al. is the lack of a preoperative assessment of quality of life, which unfortunately does not allow conclusions to be drawn regarding potential postoperative improvements. Additionally, long-term outcomes, including hernia recurrence and patient-reported quality of life, remain insufficiently explored.

A prospective cohort clinical trial is currently underway, sponsored by RWTH Aachen University in Germany, to evaluate the functionality and feasibility of using the Fasciotens^®^ Abdomen medical device in patients with open abdomen (ClinicalTrials.gov Identifier: NCT04033614). The main objective of the study is to demonstrate the effectiveness of the device in preventing fascial retraction and facilitating subsequent abdominal closure, as well as to evaluate its safety and usability in a clinical setting [37]. The I.VE.TRA Registry (Italian VErtical TRAction Registry), a national multicentre, observational and prospective study promoted by the Italian Association of Hospital Surgeons (ACOI) is currently ongoing, and our institution is also participating. The aim of the registry is to collect and analyse surgical cases of abdominal wall reconstruction for complex laparoceles treated using the IFT technique, with any surgical approach (open, laparoscopic or robotic), in the period between 1st October 2025 and 1st October 2027 [38].

Future research should focus on prospective comparative studies and the development of standardized reporting frameworks to better define the role of vertical fascial traction within existing treatment algorithms. Incorporation of patient-reported outcomes and formal economic evaluations would further strengthen the evidence base and support informed policy decisions.

## Conclusions

This narrative review suggests that vertical fascial traction using the Fasciotens^®^ system represents a valuable adjunctive option in the management of OA and complex ventral hernias. Available evidence indicates that the technology may improve rates of primary fascial closure, reduce time to closure, and limit the need for extensive reconstructive procedures in selected high-risk patients. From a clinical perspective, Fasciotens^®^ appears to address a critical unmet need by counteracting fascial retraction and preserving abdominal wall compliance in scenarios where conventional techniques often fail. The favorable safety profile reported across published studies supports its feasibility in both emergency and elective surgical settings, particularly when integrated into multidisciplinary and standardized care pathways. Although robust cost-effectiveness analyses are currently lacking, indirect economic considerations suggest that earlier closure, reduced complication rates, and decreased resource utilization may translate into meaningful organizational and financial benefits at the hospital level. These potential advantages are highly relevant in the context of increasing pressure on intensive care capacity and rising costs associated with complex abdominal wall reconstruction. Nevertheless, the current evidence remains limited by the predominance of non-comparative and low-level studies. Further prospective comparative studies and formal economic evaluations are needed to better define the role of Fasciotens^®^ within contemporary abdominal wall reconstruction strategies.

## Data Availability

The datasets generated and analyzed during the current study are available from the corresponding author on reasonable request.
